# Synthesis of 3-Alkyl Pyridinium Alkaloids from the Arctic Sponge *Haliclona viscosa*

**DOI:** 10.3390/md8030483

**Published:** 2010-03-05

**Authors:** Christoph Timm, Thorsten Mordhorst, Matthias Köck

**Affiliations:** Alfred-Wegener-Institut für Polar- und Meeresforschung in der Helmholtz-Gemeinschaft, Am Handelshafen 12, D-27570 Bremerhaven, Germany; E-Mails: CTimm@novamelt.de (C.T.); Thorsten.Mordhorst@awi.de (T.M.)

**Keywords:** 3-alkyl pyridinium alkaloids, Haliclona viscosa, synthesis, marine natural products

## Abstract

3-Alkyl pyridinium alkaloids (3-APAs) are common secondary metabolites in marine sponges of the order Haplosclerida. In recent years, our laboratory has isolated and synthesized several new members of this family such as haliclamines C–F, viscosamine, viscosaline and a cyclic monomer. All of them were isolated from the Arctic sponge *Haliclona viscosa* collected in Spitsbergen, Norway. In this article we report the syntheses of these secondary metabolites from *Haliclona viscosa* and related compounds and give a short overview of the bioactivity.

## 1. Introduction

The marine sponge *Haliclona viscosa* inhabits Arctic waters and the North Sea. In the European region it is most common around the British Isles up to Spitsbergen, Norway, but can also be found in some regions of the French and Belgian coasts. Secondary metabolites of this sponge are active in biological tests for antibacterial [1], antifungal [2], cytotoxic [2], and feeding deterrent [3] compounds.

## 2. Natural Products from *Haliclona viscosa*

A large number of 3-APAs have been isolated from marine organisms, especially from sponges of the order Haplosclerida (mainly from the genus *Haliclona*). Although 3-APAs look structurally quite simple, the structure elucidation by NMR spectroscopy is complicated by the fact that most of the methylene groups in the alkyl chains show the same chemical shift. Therefore, the 3-APAs are an ideal test case for a combined approach of NMR spectroscopy and mass spectrometry. In the NMR spectrum only the positions 1, 2, *n*−1, and *n* of the alkyl chain can be differentiated. Information about the length of the alkyl chain is only available from MS/MS spectra.

**Figure 1:**
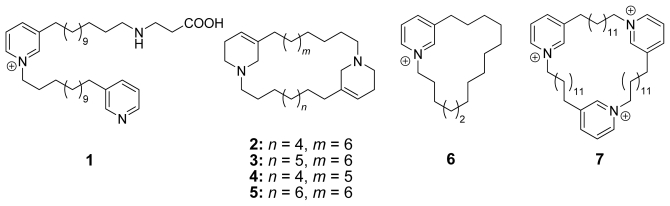
Structures of new natural products **1**–**7** isolated from *Haliclona viscosa*.

In samples of *Haliclona viscosa*, which were collected between 1999 and 2003, several new secondary metabolites – viscosaline (**1**), haliclamines C–F (**2**–**5**), the known cyclostellettamine C (Figure 4, **23**), a cyclic monomer (**6**) and the trimer viscosamine (**7**) (all Figure 1) – were isolated and identified by NMR and MS methods.

All of these compounds are characterised by six-membered azacycles, which are connected to alkyl chains at position 1 and/or 3. Regardless of the oxidation state of the six-membered ring, we classify these compounds as 3-APAs since most of the rings possess a pyridine or pyridinium structure. The 3-APAs can be divided into two main groups: (a) linear (Section 2.1) and (b) cyclic compounds (Section 2.2). These groups can be further distinguished by the number of six-membered azacycles in one molecule, e.g., monomer, dimer, trimer and so on.

### 2.1. Linear 3-APAs

**Figure 2:**
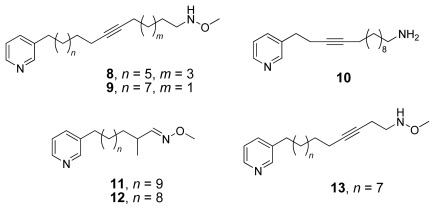
Linear and monomeric 3-alkyl pyridinium alkaloids **8**–**13**.

The majority of the isolated compounds of the 3-APA family belong to the group of the linear monomers. In comparison with other groups the linear monomers exhibit a broader constitutional variety. Their alkyl chain length varies from twelve to eighteen methylene units; double and triple bonds as well as a nitrogen based functional group are common features. Examples of linear 3-APAs are: niphatynes A (**8**) and B (**9**) [4], niphatesins A (**10**) and G (**11**) [5,6] (all isolated from *Niphates* sp.) and ikimines A (**12**) and D (**13**) [7] isolated from an unidentified sponge from Micronesia (see Figure 2).

**Figure 3:**
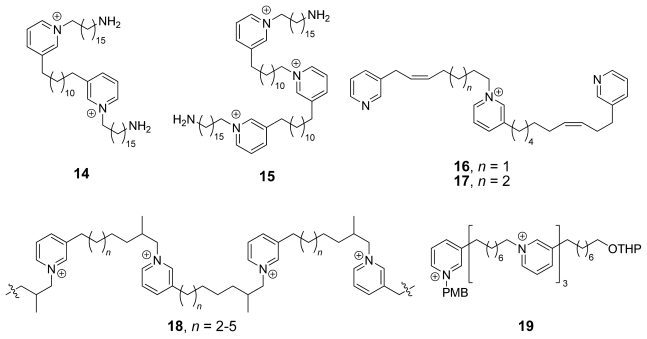
Chemical structures of linear and oligomeric 3-APAs **14**–**19**.

With the increasing number of monomeric units within the 3-APAs, the chemical diversity and the size of the attached functional groups decreases. Examples include pachychalines A (**14**) and C (**15**) [8], both isolated from *Pachychalina* sp., niphatoxins A (**16**) and B (**17**) [9], and viscosaline (**1**, Figure 1) isolated from *Haliclona viscosa* [10]. Even larger members are the halitoxins (**18**) [11], isolated from a *Haliclona* species and the synthetic linear oligomer **19** [12] (see Figure 3). The dimer viscosaline (**1**) was the first 3-APA linked to an amino acid moiety isolated from natural sources.

The connectivity of the monomeric units within the di- and trimers is not necessarily head/tail (as in the natural products from *Haliclona viscosa*), as head/head and tail/tail linked dimers are known. The linear 3-APAs that are shown in Figure 3 are either purely head/tail linked [e.g., pachychaline A (**14**) or the halitoxins (**18**)] or a mixture of head/tail and tail/tail connected units [e.g., pachychaline C (**15**) and niphatoxins A (**16**) or B (**17**)]. The synthetic isocyclostellettamines (see Scheme 7) can be regarded as head/head and tail/tail linked isomers of the naturally occuring cyclostellettamines.

### 2.2. Cyclic 3-APAs

The above-mentioned simplification of the chemical structure is also observed for cyclic 3-APAs. Next to the azacycles, double bonds are the only functionality observed in cyclic 3-APAs. A secondary metabolite isolated from *Haliclona viscosa* is the only naturally occuring cyclic monomer (**6**) which represents the smallest member of the cyclic 3-APAs macrocycles [13–15]. Prior to its isolation these molecules have been observed as side products in the synthesis of cyclostellettamines [13,14].

Dimeric macrocycles dominate the group of the cyclic 3-APAs. They lack side chains and the azacycles share the same oxidation state, but the length of the alkyl chains as well as the number of double bonds in one chain may vary. Most pyridinium salts or cyclostellettamines (A–F, **21**–**26**) [16] carry no double bonds with the exception of the dehydrocyclostellettamines D and E isolated by Fusetani *et al.* [17]. Further cyclostellettamines (G–L) were isolated from sponges of the genera *Xestospongia* [17] and *Pachychalina* [18]. The corresponding tetrahydropyridine compounds are called haliclamines (**2**–**5** and **27**, **28**) [2,19–22]. Haliclamines A (**27**) and B (**28**) have two and three double bonds within the alkyl chains [2], respectively, whereas haliclamines C–F (**2**–**5**) lack unsaturation in the alkyl chains.

**Figure 4:**
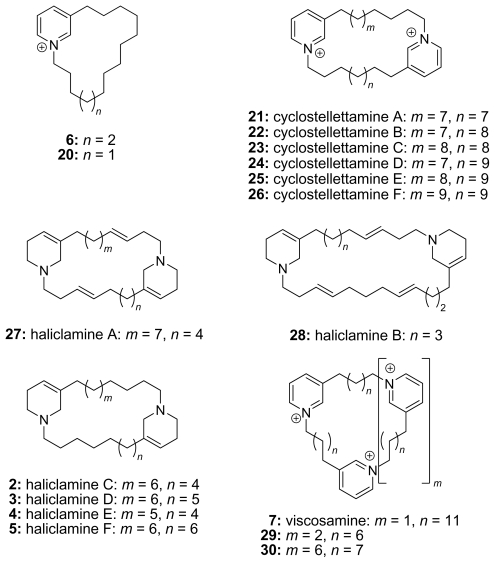
Chemical structures of cyclic 3-APAs.

There are only two reports of 3-APA macrocycles with three or more monomeric units from natural sources. Teruya *et al.* isolated a mixture of di- to hexamers of 3-dec-3-enpyridines and named them cyclohaliclonamines [23]. Our group isolated and synthesized the cyclic trimer viscosamine (**7**) [24]. The other macrocycles are synthetic compounds such as the tetrapyridinium macrocycle (**29**) [25] or even larger macrocycles (*i.e*., **30**) [14]. Representatives are shown in Figure 4.

## 3. Biological Activity

Our interest in the sponge *Haliclona viscosa* was raised some years ago during a general investigation on invertebrates from Spitsbergen in which 18 abundant sessile or slow-moving species were studied with respect to their feeding deterrence and antimicrobial activity [3,10,26–28]. The feeding deterrence was tested against the amphipod *Anonyx nugax* (a common predator in Spitsbergen) and the starfish *Asterias rubens* from the North Sea [3,26,27]. Only two of the 18 crude extracts (*Haliclona viscosa* and the actinian *Hormathia nodosa*) showed significant activity in the feeding deterrence assay. After fractionation of the crude extract of *Haliclona viscosa* only the *n*-hexane (*P* = 0.02) and *n*-butanol fractions (*P* < 0.01) were significantly deterrent against *Anonyx nugax*. The remaining water fraction caused no effect. Testing of the pure compounds revealed that only one compound from the *n*-hexane fraction (*P* < 0.01) and one compound from the *n*-butanol fraction (*P* < 0.01) were active. The *n*-butanol fraction contains the 3-APAs. The active component was identified as viscosaline (**1**) whereas haliclamine C (**2**, *P* = 0.58) and haliclamine D (**3**, *P* = 0.24) were not active. In the star fish assay only the crude extract of the sponge *Haliclona viscosa* was feeding deterrent, whereas no activity was observed for the other crude extracts.

For the investigation of the antimicrobial activity five bacterial strains were isolated from the vicinity of the sponge. The crude extract of *Haliclona viscosa* showed a very strong activity against all five bacteria [28]. Three crude extracts from the soft coral *Gersemia rubiformis*, the bryozoan *Alcyonidium gelatinosum*, and the nudibranch *Flabellina salmonacea* inhibited the growth of two bacterial strains whereas the extract of *Hormathia nodosa* showed no activity at all. The *n*-butanol fraction of *Haliclona viscosa* still showed a strong activity against all five bacteria. No activity was observed for the *n*-hexane and water fractions. The pure compounds affected only two to four out of the five bacteria. Viscosaline (**1**) showed a moderate activity against four bacterial strains whereas the halicamines C and D (**2** and **3**) showed a very strong activity against two bacteria. Haliclamine D (**3**) also showed a weak inhibition against one further bacterial strain.

The crude extracts and the four fractions (*n*-hexane, ethyl acetate, *n*-butanol, and water) of *Haliclona viscosa* (2000 and 2001) were also tested against 17 microorganisms [10]. Strong activities were only observed for the ethyl acetate and the *n*-butanol fractions from the specimen collected in 2000. From the 2001 specimen, crude extracts were strongly active against four bacterial strains (*Aquaspirillum psychrophilum*, *Microbacterium barkeri*, *Micrococcus* sp., and *Roseobacter litoralis*), and bioactivity was principally due to the *n*-butanol fraction.

Several pure compounds synthesized in our laboratory were further tested in antimicrobial assays against *Escherichia coli* tolC and *Staphylococcus aureus* and for their cytotoxicity against mouse fibroblasts L929 [15]. Monomeric linear compounds without functional groups were not active at all, whereas linear molecules with functional groups showed a moderate activity. The strongest activity was observed for cyclic compounds in all three assays.

## 4. Synthesis

The main focus of our synthetic work was the preparation of cyclic 3-APAs. The combination of different known methods generated a synthesis scheme which allowed a module-like preparation of many different structures from one alkyl pyridine precursor. The syntheses of monomeric, dimeric and trimeric cyclic 3-APAs are conducted in three steps [29]:

1. synthesis of the monomers,
2. functionalisation of the monomers, and
3. coupling and/or cyclisation.

As monomeric units we synthesized 3-ω-hydroxy alkyl pyridines with varying chain lengths. The synthesis commenced with a dicarboxylic acid **1-I**, a diol **1-II** or a bromo alcohol **1-III**, depending on their commercial availability.

**Scheme 1:**
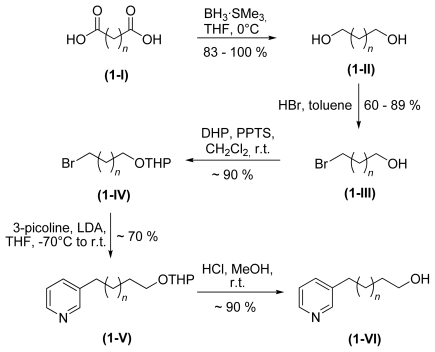
Synthesis of different 3-ω-hydroxyalkyl pyridines with *n* = 3–11.

**Scheme 2:**
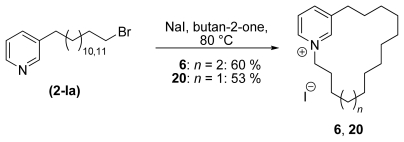
Synthesis of monomeric cyclic 3-APAs **6** and **20**.

**Scheme 3:**
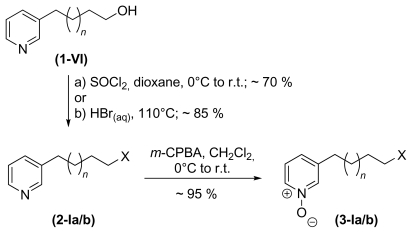
Functionalisation of 3-ω-hydroxyalkyl pyridines with *n* = 3–11 and X = Br (**a**) or X = Cl (**b**).

**Scheme 4:**
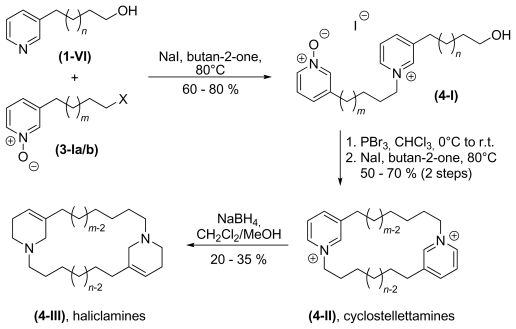
Synthesis of cyclostellettamines and haliclamines (X = Cl or Br, *n* = 3–11, *m* = *n* − 1).

Scheme 1 shows the synthetic strategy with the formation of the protected 3-alkyl pyridine **1-V** and the target molecule after cleavage of the protecting group **1-VI** [30,31]. The next step in the synthesis was the functionalisation of the monomers prior to coupling and/or cyclisation. To prepare monomeric cyclic 3-APAs, alcohol **1-VI** was converted to a 3-ω-bromo alkyl pyridine **2-Ia** (see Scheme 3). This molecule reacted intramolecularly to form the target molecules (**6** and **20**) as shown in Scheme 2. The identical reaction conditions explain the fact that monomeric cyclic 3-APAs also occur as side products within the synthesis of higher oligomeric and cyclic 3-APAs (Scheme 4, first step, reaction to **4-I**) when the ring nitrogen atom is not protected [32].

The syntheses of haliclamines and cyclostellettamines were carried out according to a method introduced by Baldwin and co-workers [32]. The monomer was activated by the conversion of the hydroxyl group to a halogenide (**2-Ia**, bromide or **2-Ib**, chloride) and protected by oxidation of the ring nitrogen atom to form *N*-oxide **3-Ia/b**, as shown in Scheme 3.

In the next step, the activated and protected molecule **3-Ia/b** was coupled with another 3-ω-hydroxyalkyl pyridine **1**–**VI**. To enhance coupling effiency during the dimerisation **3-Ia/b** was further activated by conversion to an iodide in an *in situ* Finkelstein substitution of chloride or bromide. Deprotection and activation of the resulting dimer took place in a single step prior to cyclisation. Cyclisation to form the cyclostellettamines **4-II** was carried out under pseudo high dilution conditions and required no further isolation or purification of the activated dimer. In the last step the corresponding haliclamines **4-III** were obtained by reduction with sodium borohydride (Scheme 4).

In the synthesis of 3-APA dimers and trimers, the key step is the coupling of the monomers, which must be functionalised depending on the chosen method. The most straightforward approach is the use of the pyridine nitrogen as a nucleophile in a substitution reaction, which requires an electrophilic terminal carbon of the partner monomer. Its activation was achieved *via* introduction of a good leaving group. To prevent intramolecular reactions or uncontrolled oligomerisation, a suitable protecting group for the ring nitrogen of the electrophile had to be introduced.

**Scheme 5:**
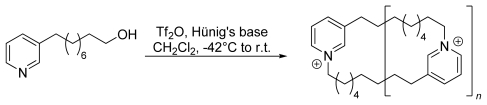
Synthesis of 3-alkyl pyridinium macrocycles by Faulkner *et al.* (*n* = 1, 2, or 3).

Faulkner *et al.* [25] reported a small scale one step/pot synthesis of cyclic 3-APAs without protecting groups using *in situ* activation of the monomers (Scheme 5). They were able to isolate monomers, dimers, trimers, and even higher oligomers *via* HPLC. In our group an analogous synthetic approach failed. We could only isolate very small quantities of dimeric 3-APAs.

Besides the nucleophilic substitution approach, Marazano *et al*. [33] used the Zincke reaction of an amine and a dinitrophenyl pyridinium salt to generate oligomeric 3-APAs. His group also reported a biomimetic synthesis in which coupling was achieved *via* a pyridine ring formation [34]. Olefin metathesis as applied by Balando and co-workers [35] and the optimized synthesis with other strategies of protection and deprotection [36] open further avenues for the generation of pyridinium macrocycles.

**Scheme 6:**
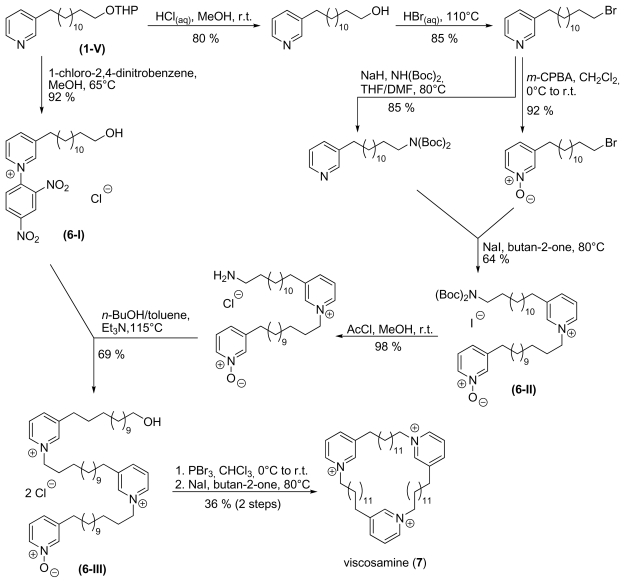
Synthesis of viscosamine (**7**).

In order to increase the number of monomeric units within the macrocycles, the previously described synthesis of cyclostellettamines was expanded as shown in Scheme 6.

Activation and deprotection in one step prior to cyclisation limits the synthesis by Baldwin *et al.* [32] to dimers. By using an orthogonally activated monomer, it was possible to use the advantages of the dimer synthesis and insert an additional monomeric unit. Dimer **6-II** was prepared from *N*-oxide **3-Ib** and a bis-protected alkylpyridineamine like Baldwin’s cyclostellettamine precursors (**4-I**). Unlike these dimers, the amino dimer **6-II** can – after cleavage of the Boc-protection group – react with hydroxy Zincke-salt **6-I**, which was prepared in one step from protected 3-ω-hydroxyalkyl pyridine **1-V**. The linear trimer **6-III** was then deprotected, activated and cyclised to provide the target molecule viscosamine (**7**). By combining the approaches of Baldwin *et al.* [32] and Marazano *et al.* [33] we were able to prepare viscosamine (**7**) as a trimer and save one step compared to Marazano’s method. It also reduced the number of steps with charged oligomers, thus facilitating the purification.

**Scheme 7:**
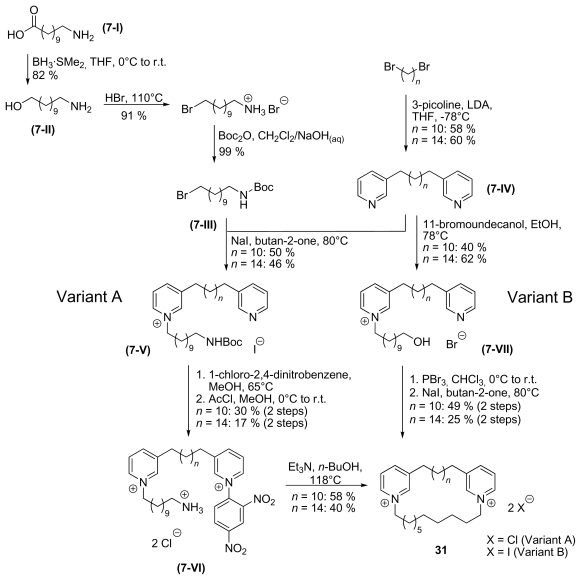
Synthesis of isoscyclostellettamines.

For the synthesis of isocyclostellettamines (Scheme 7), the synthetic strategy had to be modified. Two variants for the preparation of isocyclostellettamines were established. Variant A starts with an ω-aminocarbonic acid **7-I** that was first reduced to the alcohol **7-II** and then protected to give **7-III**. By reacting **7-III** with an 1, ω-dipyridine alkane **7-IV**, an unsymmetric and mono-alkylated 1, ω-dipyridine alkane **7-V** was formed. Cyclisation to the target molecule was achieved *via* Zincke-salt **7-VI**. Variant B started with 1, ω-dipyridine alkane **7-IV** that was directly alkylated with 11-bromoundecanol to form 1, ω-dipyridine alkane **7-VII**, from which the target molecule was directly accessed. Scheme 7 shows the difference of the synthetic strategies *via* a Zincke-salt or *via* a nucleophilic substitution by the pyridine nitrogen.

Variant B has the advantage that it is shorter than variant A and the bromoalcohol for the first alkylation is commercially available. Furthermore the overall yield through both variants is in variant B (10% over 4 steps) almost three times higher than in variant A (4% over 7 steps). But variant A underlines the good applicability of the Zincke-salt for the synthesis with the disadvantage of an additional step and lower yields.

**Scheme 8:**
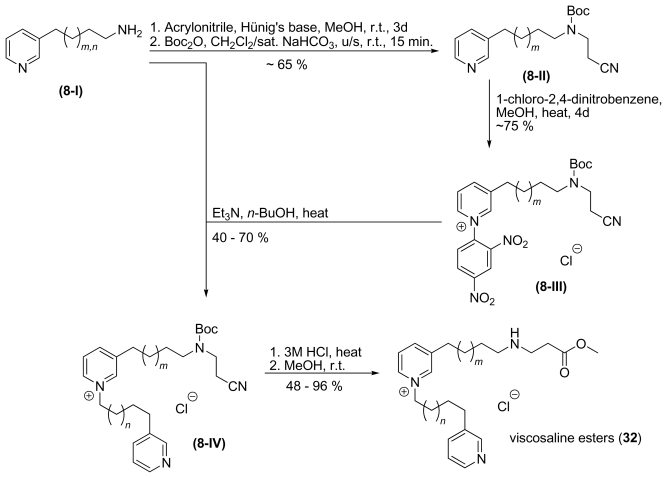
Synthesis of viscosaline esters **32** with *m*, *n* = 9–11.

The synthesis of viscosaline (**1**) as a functionalized linear 3-APA was previously discussed as a module-like synthesis in order to prepare various analogs with different amino acid residues in the terminal position. This approach failed in numerous ways. Neither reductive amination of β-Ala derivatives [37] nor their alkylation with halogenalkyl pyridines [38] led to the desired molecules in sufficient yields. Further experiments lead to a single synthesis for a β-alanine moiety. Similar to the synthesis of viscosaline (**1**) by Baldwin *et al.* [39], the amino acid moiety is introduced *via* an aza- Michael reaction. A 3-ω-aminoalkyl pyridine **8-I** was treated with acrylonitrile and Hünig’s base and subsequently protected to form the alkylated and protected amine **8-II**. Since **8-II** is prepared from amino alkyl pyridine **8-I**, the utilisation of 1-chloro-2,4-dinitrobenzene was an adequate and advantageous way to activate the pyridine ring by conversion to Zincke-salt **8-III**, which was coupled to 3-ω-aminoalkyl pyridine **8-I** at the ring nitrogen to form salt **8-IV**. Finally, the remaining protecting group was removed and the nitrile moiety was oxidized and immediately esterified in the presence of methanol to the viscosaline esters (**32**), as shown in Scheme 8.

## 5. Conclusions

**Figure 5:**
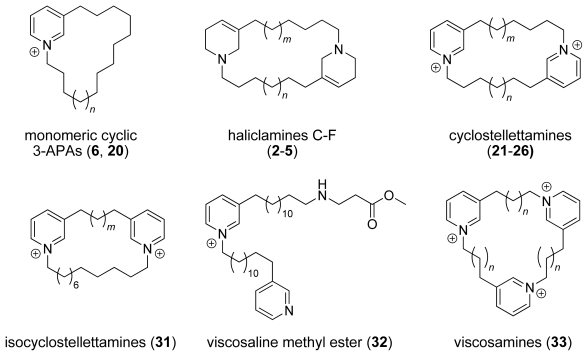
Chemical structures of the synthesized compounds (no anions shown).

During our project a series of natural compounds and related derivatives were synthesized. Our synthetic effort started with the synthesis of cyclostellettamines and haliclamines according to a methodology described by Baldwin and co-workers. In the synthesis of viscosamine (**7**) we had to overcome the limitation to dimeric structures of Baldwin’s approach by combining it with Marazano’s application of the Zincke reaction. Cyclic 3-APAs with one (**6**, **20**) or three (**33**) monomeric units with alkyl chains of different length can thus be prepared, opening a way to a library of different 3-APAs for further experiments. The synthesis of isoscyclostellettamines **31** provides access to material which can be used to deepen the understanding of the biological activities of 3-APAs. In the synthesis of viscosaline (**1**), we experienced unexpected problems in the preparation of the amino acid bearing alkyl pyridine. Further experiments should lead to a more general synthesis which shall provide 3-APAs with various amino acid functionalities. Figure 5 summarizes the general structures of the compounds we have synthesized so far and originally isolated from the Arctic sponge *Haliclona viscosa*.
